# Classifying *Escherichia coli*

**DOI:** 10.3201/eid1208.051654

**Published:** 2006-08

**Authors:** Dennys M. Girão, Valéria B.C. Girão, Kinue Irino, Tânia A. Tardelli Gomes

**Affiliations:** * Universidade Federal do Rio de Janeiro, Rio de Janeiro, Brazil;; † Universidade de São Paulo, São Paulo, Brazil;; ‡ Instituto Adolfo Lutz, São Paulo, Brazil;; § Universidade Federal de São Paulo, São Paulo, Brazil

**Keywords:** Escherichia coli, atypical EPEC, localized adherence, aggregative adherence properties, attaching, effacing, diagnosis, epidemiology, diarrhea, letter

To the Editor: Enteropathogenic Escherichia coli (EPEC), 1 of the 6 pathotypes of diarrheogenic E. coli (DEC), promotes attaching-effacing lesions in eukaryotic cells. These lesions are mediated by intimin, an outer membrane adhesive protein encoded by the eae (E. coli attaching-effacing) gene (*1*). EPEC is currently subdivided into typical and atypical subgroups. While typical EPEC carry the EPEC adherence factor plasmid (pEAF) that encodes the bundle-forming pilus (BFP) and a complex regulator of various virulence genes (Per) (*1*), atypical EPEC is devoid of pEAF (or does not express a functional BFP) (1,2). Typical EPEC expresses the localized pattern of adherence (LA), which is characterized by compact bacterial clusters on HeLa and HEp-2 cells (*1*). Conversely, atypical EPEC most often expresses the LA-like pattern (with loose bacterial clusters) or adherence patterns of other DEC pathotypes (*2*).

Enteroaggregative E. coli (EAEC), another DEC pathotype, is identified by the characteristic aggregative pattern of adherence (AA) in HeLa/HEp-2 cells; bacteria attach in aggregates to cell surfaces as well as around cells (*1**,**3*). EAEC colonizes the intestinal mucosa, forming a thick biofilm that favors prolonged colonization and induces malnutrition (*1**–**3*). Actually, this pathotype is heterogeneous regarding the presence of putative virulence genes and has recently been subgrouped into typical and atypical EAEC, which carry and lack AggR (a global regulator of EAEC virulence), respectively (*1**,**3*).

We recently conducted a study at the Instituto de Puericultura e Pediatria Martagão Gesteria in Rio de Janeiro, Brazil, on the etiology of diarrhea affecting children of low socioeconomic status (V.B.C. Girão et al., unpub. data). In the study, all E. coli isolates were analyzed regarding their adherence patterns in HeLa cells and the presence of specific virulence genes of the DEC pathotypes, according to previously reported methods (*4**,**5*). Among 481 children (<2 years old) with diarrhea who were examined, 16 (3.3%) carried E. coli strains that co-expressed LA and AA (LA/AA), a phenotype not found among strains of 99 control children without diarrhea at the same hospital. The LA/AA phenotype was confirmed in individual colonies of each strain as well as in HEp-2 cells. In both cell lineages, prolonged assays (6 hours) showed that a mature biofilm that masked the LA phenotype had developed.

Although LA/AA co-expression in some human E. coli has been previously reported by Bouzari et al. (*6*), further information on these isolates is lacking. Moreover, since the expression of LA and AA is used to classify fecal E. coli as typical EPEC and EAEC (*1**,**3*), respectively, the classification of such strains within the DEC pathotypes is difficult. To determine their most appropriate classification, we further characterized the 16 LA/AA strains of our collection (Table). Colony hybridization assays used to search for additional E. coli virulence genes (bfpA, perA, E-hly, daaC, cdt, cnf, hly, aggR, aggC, aafC, aap, shf, irp2, pet, pic, astA, pap, afa, sfa, efa, paa, saa, enfA) (*1**,**3**–**5**,**7*) showed that all strains carried eae, bfpA, and perA, and 13 also carried the EAF sequence (a cryptic pEAF marker). Less commonly found genes were paa, shf, irp2, astA, and efa, and the remaining genes were absent. BFP expression was confirmed in all strains by immunoblot, and positivity in the fluorescent actin staining assay (*8*) demonstrated that they can produce attaching/effacing lesions. PCR analysis of 4 (α, β, γ, and δ) (*9*) of at least 10 recognized intimin subtypes (*1*) showed that subtype δ was the most frequent. Serotyping (*5*) identified at least 10 distinct serotypes among the 16 strains, which demonstrated that they do not make up a single clone. Two serotypes (O119:H6 and O142:H6) are commonly found among typical EPEC (*2*). Certain typical and atypical EPEC serotypes have been associated with distinct intimin subtypes (*9*). Likewise, our LA/AA strains of the same serotype carried the same intimin subtype. Recently, Carvalho et al. (*10*) detected LA/AA expression in 4 of 21 eae-positive E. coli strains isolated from monkeys with diarrhea. All 4 strains expressed BFP and lacked the EAF sequence; as in our study, 1 belonged to serotype O142:H6 and carried intimin α.

**Table:** Genotypic and phenotypic properties of 16 *Escherichia coli* strains that co-express localized and aggregative patterns of adherence*

Strain	Virulence genes	Intimin subtype	Serotype
98180	*eae*, *bfp*A, EAF, *per*A, *paa*	δ	O2:H45
99137	*eae*, *bfp*A, EAF, *per*A, *paa*	δ	O2:H45
21153	*eae*, *bfp*A, EAF, *per*A, *shf*	δ γ†	O55:H51
21187	*eae*, *bfp*A, EAF, *per*A, *shf*	δ γ†	O55:H51
99253	*eae*, *bfp*A, EAF, *per*A, *shf*	δ γ†	O55:HNM
99329	*eae*, *bfp*A, EAF, *per*A, *shf*	δ γ†	O55:HNM
99197	*eae*, *bfp*A, EAF, *per*A	β δ†	O119:H6
98288	*eae*, *bfp*A, EAF, *per*A, *irp2*	β	O119:H6
22622	*eae*, *bfp*A, EAF, *per*A, *irp2*	β	O119:H6
99336	*eae*, *bfp*A, *per*A, *paa*, *astA*, *efa*	α	O142:H6
98351	*eae*, *bfp*A, EAF, *per*A, *astA*	γ	O145:H45
22652	*eae*, *bfp*A, EAF, *per*A, *irp2*	(–)	O178:H33
99245	*eae*, *bfp*A, *per*A, *paa*, *efa*, *ast*A	α	ONT:H6
98025	*eae*, *bfp*A, *per*A, *paa*	α	ONT:H6
98366	*eae*, *bfp*A, EAF, *per*A, *efa*	β	ONT:H7
22150	*eae*, *bfp*A, EAF, *per*A, *astA*	α	ONT:H10

*Isolated from children with diarrhea. EAF, enteropathogenic *E. coli* adherence factor; –, nontypable with the primers tested. †Intimin types undetermined because amplification products of the expected size were obtained with 2 intimin pairs of primers.

E. coli classification within the DEC pathotypes has epidemiologic and clinical implications for managing diarrheal diseases. However, finding E. coli isolates that co-express LA/AA reiterates the difficulty of assigning bacteria to groups on the basis of their adherence phenotype or genotype (particularly when based on mobile genetic elements). Since our analysis with molecular methods showed that these strains carry more characteristics of typical EPEC and lack the AggR regulon, we propose that they be classified as typical EPEC. Typical EPEC are recognized as pathogens, whereas atypical EAEC are not (*3*). In addition, the ability to simultaneously induce attaching/effacing lesions and biofilm production may increase the potential of these strains to cause diarrhea and prolong bacterial residence in the intestines, thus worsening malnutrition in the patient.
